# Study protocol of a multicenter randomized controlled trial comparing the effectiveness of group and individual internet-based Mindfulness-Based Cognitive Therapy with treatment as usual in reducing psychological distress in cancer patients: the BeMind study

**DOI:** 10.1186/s40359-015-0084-1

**Published:** 2015-08-13

**Authors:** F. R. Compen, E. M. Bisseling, M. L. Van der Lee, E. M. M. Adang, A. R. T. Donders, A. E. M. Speckens

**Affiliations:** Department of Psychiatry, Radboud University Nijmegen Medical Centre for Mindfulness, Postbus 9101, 6500 HB Nijmegen, The Netherlands; Scientific Research Department, Helen Dowling Institute, Centre for Psycho-Oncology, Bilthoven, The Netherlands; Department for Health Evidence, Radboud University Nijmegen Medical Centre, Nijmegen, The Netherlands

**Keywords:** Mindfulness-based cognitive therapy, Cancer, Distress, E-health, Internet-based, Randomized controlled trial

## Abstract

**Background:**

Mindfulness-based interventions have shown to reduce psychological distress in cancer patients. The accessibility of mindfulness-based interventions for cancer patients could be further improved by providing mindfulness using an individual internet-based format. The aim of this study is to test the effectiveness of a Mindfulness-Based Cognitive Therapy (MBCT) group intervention for cancer patients in comparison with individual internet-based MBCT and treatment as usual (TAU).

**Methods/Design:**

A three-armed multicenter randomized controlled trial comparing group-based MBCT to individual internet-based MBCT and TAU in cancer patients who suffer from at least mild psychological distress (Hospital Anxiety and Depression Scale (HADS) ≥ 11). Measurements will be conducted prior to randomization (baseline), post-treatment and at 3 months and 9 months post-treatment. Participants initially allocated to TAU are subsequently randomized to either group- or individual internet-based MBCT and will receive a second baseline measurement after 3 months. Thus, the three-armed comparison will have a time span of approximately 3 months. The two-armed intervention comparison includes a 9-month follow-up and will also consist of participants randomized to the intervention after TAU. Primary outcome will be post-treatment psychological distress (HADS). Secondary outcomes are fear of cancer recurrence (Fear of Cancer Recurrence Inventory), rumination (Rumination and Reflection Questionnaire), positive mental health (Mental Health Continuum – Short Form), and cost-effectiveness (health-related quality of life (EuroQol –5D and Short Form-12) and health care usage (Trimbos and iMTA questionnaire on Costs associated with Psychiatric illness). Potential predictors: DSM-IV-TR mood/anxiety disorders (SCID-I) and neuroticism (NEO-Five Factor Inventory) will be measured. Mediators of treatment effect: mindfulness skills, (Five-Facets of Mindfulness Questionnaire- Short Form), working alliance (Working Alliance Inventory) and group cohesion (Group Cohesion Questionnaire) will also be measured.

**Discussion:**

This trial will provide valuable information on the clinical and cost-effectiveness of group versus internet-based MBCT versus TAU for distressed cancer patients.

**Trial registration:**

Clinicaltrials.gov NCT02138513. Registered 6 May 2014.

## Background

Cancer is a major health care challenge. Cancer causes more than a quarter of all deaths in OECD countries with more than 5 million new cases diagnosed every year, averaging about 261 cases per 100 000 people (OECD Health Policy Studies - Cancer Care: Assuring quality to improve survival. http://www.oecd.org/els/health-systems/Focus-on-Health_Cancer-Care-2013.pdf. Accessed May 7th 2015). In the Netherlands it is expected that the incidence of cancer will increase with more than 40 % between 2007 and 2020 (KWF Kankerbestrijding 2011). These numbers indicate that we are looking at a steadily increasing number of patients who will have to cope with cancer in the near future.

Living with cancer is a psychological burden. In a review of the prevalence of depression, anxiety and adjustment disorders in cancer patients in both palliative and non-palliative settings it was found that about one third of all patients suffer from a mood disorder in the first five years after diagnosis (Mitchell et al. 2011). A recent epidemiological survey based on more than 2000 structured clinical interviews across major tumor entities found the most prevalent mental disorders to be anxiety (11.5 %) adjustment (11.1 %) and depressive disorders (6.5 %) (Mehnert et al. 2014). Considering the rising prevalence of people living with cancer, the absolute number of cancer patients in need of psychological treatment is expected to increase. Addressing this increasing need calls for effective, widely available and accessible psychological treatment.

In recent years, many studies have assessed the effect of mindfulness-based interventions for cancer patients. Mindfulness is defined as intentionally paying attention to moment-by-moment experiences in a non-judgmental way (Segal et al. 2013). Mindfulness-Based Stress Reduction (MBSR) (Kabat-Zinn 1982) and Mindfulness-Based Cognitive Therapy (MBCT) (Teasdale et al. 2000), the latter developed specifically to prevent relapse in depression, are protocols designed to teach the cultivation of mindfulness. In a review of 22 studies, mindfulness-based interventions were found to be moderately effective in reduction of symptoms of anxiety and depression in cancer patients (Piet et al. 2012). Recently, another randomized controlled trial (RCT) showed that mindfulness-based treatment was superior to both supportive-expressive group therapy and a 1-day stress management condition in improving a range of psychological outcomes in a sample of 271 distressed breast cancer survivors (Carlson et al. 2013). Although any follow-up results should still be considered preliminary, the recent review indicates that effect sizes (ES) at follow-up were significant with small to moderate ESs for nonrandomized studies and small ESs for RCTs.

Psychological treatment for cancer patients implies treatment for people who have difficulty with travelling due to cancer -related impairments or fatigue. Also, treatment scheduling should be flexible, allowing for adaptation to individual circumstances, for example ongoing radio- or chemotherapy. Taking this into account, internet-based treatment might hold promise to address these problems. A recent review concludes that guided internet-based Cognitive Behavioural Therapy (CBT) “appears to be a promising and effective treatment for chronic somatic conditions to improve psychological and physical functioning and disease-related impact” (Van Beugen et al. 2014). In addition to its clinical effectiveness, research also suggests evidence for the cost-effectiveness of internet-based CBT for somatic populations (Andersson et al. 2011; Van Os-Medendorp et al. 2012).

Literature on the effectiveness of internet-based mindfulness treatment is still scarce. There are a few studies in non-clinical populations which show that internet-based mindfulness treatment resulted in an improvement of mindfulness skills and reduction of perceived distress (Cavanagh et al. 2013; Morledge et al. 2013; Krusche et al. 2012). Recently, encouraging evidence was presented for the feasibility and efficacy of internet-based mindfulness treatment in a study of 62 underserved and distressed cancer patients (Zernicke et al. 2014). Compared to treatment as usual (TAU) patients reported an increase of mindfulness and a reduction of depressive and stress symptoms. This provides preliminary evidence for the effectiveness of internet-based mindfulness treatment compared to TAU.

Direct comparisons of internet-based mindfulness treatment to existing group treatments for distressed cancer patients are absent, let alone follow-up comparisons. One of the biggest challenges in internet intervention research is low treatment adherence (Wangberg et al. 2008) which affects treatment effectiveness (Eysenbach 2002). A recent study of internet-based MBCT for treatment of chronic cancer-related fatigue using a treatment format similar to ours indicated a non-adherence rate of 38 %, which is higher than in comparable face-to-face interventions (Bruggeman-Everts et al. 2015). The current trial will provide the first description of the relative long-term effectiveness of group- compared to internet-based MBCT by including a follow-up measurement up to 9 months post-treatment and keeping close track of treatment adherence in both intervention arms.

Thus, it is unknown whether internet-based MBCT has similar effectiveness as group-based MBCT in alleviating distress in cancer. Therefore, we primarily compare post-treatment psychological distress between group-based and internet-based MBCT. Also, effectiveness in reducing psychological distress up to nine months post-treatment will be compared between group- and internet-based MBCT. Moreover, we would like to determine whether the two interventions could reduce fear of cancer recurrence and rumination. Also, at the other end of the psychological spectrum, both group- and internet-based MBCT might be able to improve positive mental health in cancer patients compared to TAU. Furthermore, alongside the clinical trial, cost-effectiveness of both MBCT interventions compared to TAU will be determined. We expect both interventions to be cost-effective compared to TAU.

We do not expect all individuals to benefit similarly from the two interventions. Therefore, studying predictors of each intervention’s effect potentially enables us to determine who benefits most from what treatment – group-based or internet-based MBCT. In this study we would like to explore two possible predictors: the presence/absence of a DSM-IV-TR mood/anxiety disorder and the personality trait neuroticism.

Research on mindfulness-based interventions for cancer patients has focused on the prevalence and treatment of distress rather than psychiatric disorders. Not much is known on the effectiveness of MBCT in oncology patients suffering from a mood and/or anxiety disorder as opposed to patients suffering from distress. We are interested to see if the presence of a psychiatric disorder is a better predictor of treatment outcome than psychological distress.

Moreover, previous research has shown that a high score on neuroticism has a negative effect on (group) psychotherapy outcome (Ogrodniczuk et al. 2003). This study aims to explore the hypothesis that higher neuroticism at baseline has a negative predictive value for the primary outcome measure and to explore possible differences in treatment outcome between group- and internet-based MBCT.

As it is known that mindfulness skills mediate the relationship between mindfulness practice and improvements in psychological symptoms (e.g. Gu et al. 2015), we hypothesize that the improvement on the Hospital Anxiety and Depression Scale (HADS) in the MBCT intervention arms is mediated by mindfulness skills. Moreover, weekly measurements (MAAS and I-PANAS-SF) will be used to test the hypothesis that an increase in mindfulness skills antedates changes in affect during the intervention.

One of the differences between face-to-face and online treatment is the relationship with the therapist. Working alliance, or therapeutic alliance, is a long-recognized concept in psychotherapy research. Although it is known that a working alliance is realizable in internet-based therapy (Cook and Doyle 2002), little is known about the possible difference in working alliance between group- and internet-based MBCT. We would be interested to see if working alliance mediates the relationship between intervention and outcome in both interventions.

The relationship with both the therapist and other group members in group-based treatment, or group cohesion, is often considered to be one of the most important contributors to positive treatment effect in group therapy. The current study aims to assess whether group cohesion mediates the relationship between the group-based MBCT intervention and outcome.

In conclusion, the primary aim of this study is to compare the effectiveness of group- and internet-based MBCT to TAU to reduce distress in cancer patients after treatment. Secondary outcome measures will be fear of cancer recurrence, rumination, and positive mental health. In addition, possible effect predictors (DSM-IV-TR mood/anxiety disorder and neuroticism) and mediators (mindfulness skills, working alliance, group cohesion) of treatment outcome will be explored. In order to determine the long-term stability of intervention effects, assessments will take place 3 and 9 months post-treatment. Alongside the clinical trial, the cost-effectiveness of both MBCT interventions compared to TAU will be determined. As far as we know, this is the first direct comparison between group-based MBCT, internet-based MBCT and TAU.

## Methods/Design

### Study design

This study is a multicenter, parallel group randomized controlled trial. Participants are randomized to group-based MBCT, internet-based MBCT or TAU. Participants initially randomized to TAU are subsequently randomized to either group- or internet-based MBCT which participants receive after a waiting-list period of three months. During the waiting-list period, participants know which treatment they will receive after the waiting list and participants are allowed to receive care as usual, except for any mindfulness-based intervention. The study protocol has been approved by our ethical review board (CMO Arnhem-Nijmegen) and is registered under number 2013/542.

### Setting

The group MBCT is provided at the Radboud University Medical Centre in Nijmegen, the Jeroen Bosch Hospital in ‘s Hertogenbosch and at four mental health institutes specialized in psycho-oncology (Helen Dowling Institute (Bilthoven), Ingeborg Douwes Centrum (Amsterdam), De Vruchtenburg (Leiden), Het Behouden Huys (Haren)). The internet-based MBCT has been developed with, protected and hosted by IPPZ, a commercial e-health company in the Netherlands. Patients receive an invitational e-mail with the conditions of use. The internet-based MBCT is accessed using a personal double-step-verification- protected webpage on the participants’ own personal computer, mobile phone or tablet device.

### Study population

Inclusion criteria of the study are a) a cancer diagnosis, any tumor or stage b) a score of 11 or higher on the Hospital Anxiety and Depression Scale (HADS), c) computer literacy and internet access d) a good command of the Dutch language and e) willingness to participate in either MBCT intervention. Exclusion criteria are a) severe psychiatric morbidity such as suicidal ideation and/or psychosis b) change in psychotropic medication dosage within a period of three months prior to baseline c) current or previous participation in a mindfulness-based intervention (>4 sessions of MBCT or MBSR).

### Procedure

Participants are recruited in aforementioned participating centers and recruited via social media, patient associations and advertorials in local newspapers. Patients who are interested in participation can enroll themselves at our website (www.bemind.info) at which point they complete the HADS. Patients with a score of 11 or higher are contacted by telephone by one of the researchers. During this call more information about the study is provided and eligible patients are invited for a research interview. The subsequent research interview is conducted face-to-face or by telephone depending on participant preference. Written informed consent, demographic and clinical characteristics are obtained on paper via regular mail. Subsequently the Structured Clinical Interview for DSM-IV-TR Axis-I disorders (SCID-I) is administered to diagnose possible mood/anxiety disorders and the Trimbos and iMTA questionnaire on Costs associated with Psychiatric illness (TiC-P) to assess medical and productivity loss costs. The participant completes the remainder of the (self-report) questionnaires online.

### Randomization

Randomization is stratified for setting and minimized for a) gender, b) stage of disease (curative *versus* palliative) and c) type of cancer (breast cancer *versus* other). Randomization is computerized using a randomization website specifically designed for the current study. Randomization is conducted by one of the researchers (EB) who is not involved in the follow-up assessments.

### Follow-up assessments

Follow-up assessments take place directly post-treatment and at three and nine months follow-up. The follow-up assessments are similar to the baseline assessment: participants are contacted by telephone in order to re-administer the SCID-I and the TiC-P and participants receive an online survey with the self-report scales. In case of dropout, the researcher tries to contact the participant at least three times to complete the outcome measures and to identify the main reason for dropout.

### Intervention

The MBCT curriculum used in both group and internet-based MBCT interventions is primarily based on the MBCT program by Segal, Williams and Teasdale (Segal et al. 2013). The program was adapted to the oncology patient in terms of tailoring psycho-educative elements to themes relevant to the cancer patient (e.g. cancer-related fatigue) and adapted movement exercises (for patients suffering from edema). In both conditions, participants receive guided mindfulness meditation exercises for home practice and a reader with home practice instructions and background information.

The group-based MBCT curriculum consists of 8 weekly 2,5 h group sessions, a silent day between session six and seven and home practice assignments of about 45 min, 6 days per week (see Table 1). During the weekly sessions the teacher guides different mindfulness exercises and introduces new exercises, and home practice assignments are discussed.

**Table 1:** MBCT curriculum content

Theme of session	Meditation exercise	Didactic teaching	Homework
1. The automatic pilot	- Body scan	- Intention of participating	- Bodyscan
			- Mindful eating
		- Raisin exercise	- Mindful routine activity
2. Dealing with barriers	- Body scan	- Observation exercise “walking through the streets”	- Bodyscan or mindfulness of the breath
		- Mindfulness of the breath	- Positive experiences diary
			- Mindful routine acitivity
3. Mindfulness of the breath	- Movement exercises lying down	- 3-min breathing space	- Body scan or movement exercises
	- Mindfulness of the breath and body		- Negative experiences diary
			- 3-min breathing space three times a day
4. Staying present	- Sitting meditation	- Psycho-education “reacting/responding stress”	- Sitting meditation or walking meditation or movement exercises
	- Walking meditation		
			- Stress diary
			- 3-min breathing space
			- Walking meditation
5. Allowing	- Sitting meditation	- Psycho-education “anxiety, anger and depression, helping and non-helping thoughts”	- Sitting meditation
	- Walking meditation		- Mindful communication exercise
			- 3-min breathing space
			- Walking meditation
6. Mindful communication	- Movement exercises standing up	- Psycho-education “communication”	- Sitting meditation, movement exercises or body scan
	- 3-min breathing space in stressful situations	- Nonverbal (Aikido) and verbal (Deeply listening) communication exercises	- 3-min breathing space
			- Walking meditation
Silence day	- Various exercises		
	- Silent lunch and tea breaks		
7. Taking care of yourself	- Sitting meditation, open awareness	- Energy balance and relapse prevention	- Mindful exercise at will
	- 3-min breathing space		- Relapse prevention plan
			- 3-min breathing space
8. From stress to inner strength	- Body scan	- Training evaluation and glance at the future	

The internet-based MBCT intervention is similar to group MBCT in curriculum content, but different in delivery. Participants in the internet-based MBCT intervention log in on a secure personal webpage where all content relevant to that week’s session can be downloaded. Participants are asked to read the weekly information and do the mindfulness exercises and write down their experiences in their personal log. They are encouraged to correspond with their personal teacher about their practice experiences via a secure, integrated mailing system. The teacher replies to this log on a predetermined day of the week and guides the participant through the curriculum. Participants can continue with next weeks’ session only after registering their experiences in their log for the previous week. Participants are encouraged to follow the intervention within the nine-week structure. However, the teacher can decide to extend this period in case of illness or holidays.

All teachers fulfill the advanced criteria of the Association of Mindfulness Based Teachers in the Netherlands and Flanders) which are in concordance with the UK Mindfulness-Based Teacher Trainer Network Good Practice Guidelines for teaching mindfulness-based courses (UK Network for Mindfulness-Based Teachers Good practice guidelines for teaching mindfulness-based courses. http://mindfulnessteachersuk.org.uk/pdf/teacher-guidelines.pdf. Accessed 31st of March 2015), including a minimum of 150 h of education in MBSR/MBCT background and theory, training in teaching formal and informal meditation practices, psycho-education and inquiry, supervision and giving an MBSR or MBCT course including a reflection report, b) relevant professional training, c) minimum of three years of practicing meditation regularly and attending retreats, d) having attended MBSR/MBCT as a participant, e) continued training and f) giving a minimum of two courses per two year. Three full-day plenary supervision meetings are held during the intervention phase of the trial, consisting of mindfulness practices, workshops, small group teachings and plenary discussions about difficulties or practical issues. All teachers are involved in both group and internet-based MBCT. Teachers without prior internet-based MBCT experience are provided with guidelines and supervised by more experienced internet-based MBCT teachers.

In the group-based MBCT condition, sessions are videotaped to evaluate teacher competence and protocol adherence using the Mindfulness-Based Interventions - Teachers Assessment Criteria (MBI-TAC) (Crane et al. 2012). The MBI-TAC was translated to Dutch using the guidelines of the International Test Commission (Hambleton 1994). Group-based MBCT participants are requested to complete the same form for their teachers’ competence. As the MBI-TAC is not applicable to internet-based treatment and there are currently no other ways to evaluate teacher competence in internet-based mindfulness treatment, teacher competence will not be assessed in the internet-based condition using a standardized measurement.

## Primary outcome measure

For a measurement scheme we refer to Table 2. The primary outcome measure is the post-treatment total score on the HADS, a 14-item self-report screening scale that was originally developed to indicate the possible presence of anxiety and depressive states in the setting of a medical outpatient clinic (Zigmond and Snaith 1983; Spinhoven et al. 1997). As earlier research in a palliative setting suggested the total HADS score should be used, this score will be used rather than individual depression and anxiety subscales (Le Fevre et al. 1999). The HADS shows good psychometric properties in the general medical population, including oncology patients in palliative phase (Akechi et al. 2006). Internal consistency as measured with Cronbach’s α varied from .84 to .90 (Spinhoven et al. 1997; Bjelland et al. 2002). Test-retest reliability was good as Pearson’s r > .80 were obtained (Spinhoven et al. 1997; Herrmann 1997).

**Table 2:** Measurement scheme

Variable goal	Measure	Target	Screening	T0	During	T0b (TAU only)	T1	T2	T3
Primary outcome	HADS	Psychological distress	x	x		x	x	x	x
Secondary outcomes	FCRI	Fear of cancer recurrence		x		x	x	x	x
	RRQ	Rumination Reflection Questionnaire		x		x	x	x	x
	MHC-SF	Mental Health Continuum – Short Form		x		x	x	x	x
Effect predictors	SCID	DSM-IV Axis I disorders		x		x	x	x	x
	NEO-FFI	Personality dimensions		x					x
Process measures	FFMQ-SF	Mindfulness skills		x		x	x	x	x
	WAI	Working alliance			x				
	GCQ	Group cohesion			x				
	MAAS	Mindfulness skills			x				
	I-PANAS-SF	Mood			x				
	Calendar	Mindfulness adherence			x				
Cost-effectiveness	TiC-P	Health care costs and productivity		x		x	x	x	x
	EQ-5D	Health-related quality of life (preference-based)		x		x	x	x	x
	SF-12	Health-related quality of life (general health profile)		x		x	x	x	x

## Secondary outcome measures

*Fear of cancer* recurrence is assessed with the Fear of Cancer Recurrence Inventory (FCRI; (Simard and Savard 2009)). This 42-item 4-point Likert scale questionnaire has been found to have a robust factor structure with Cronbach’s α = 0.75 to 0.91 across subscales and test-retest reliabilities over a two-week interval of 0.58 to 0.83 across subscales. The FCRI is positively associated with other measures of anxiety symptoms, intrusive thoughts and avoidance and negatively associated with quality of life in a large sample of cancer patients (Simard and Savard 2009).

*Rumination* is measured by the rumination subscale of the Rumination and Reflection Questionnaire (RRQ; (Trapnell and Campbell 1999)). Subjects rate their level of agreement of disagreement on a five-point rating scale (e.g., “I always seem to be re-hashing in my mind recent things I’ve said or done”). The Dutch version has Cronbach’s alphas ranging between .88 and .93 (Luyckx et al. 2008).

*Positive mental health* is measured by the Mental Health Continuum-Short Form (MHC-SF; (Keyes 2005)) which comprises 14 items, representing various feelings of well-being in the past month rated on a 6-point Likert scale (never, once or twice a month, about once a week, two or three times a week, almost every day, every day). The MHC-SF contains three subscales: emotional, psychological and social well being. The short form of the MHC has shown excellent internal consistency (> .80). The test-retest reliability of the MHC-SF over three successive 3 month periods was .68 and the 9 month test-retest in a Dutch sample was .65 (Lamers et al. 2011).

Data on medical and societal costs and data on health-related quality of life are collected to conduct the cost-effectiveness – analysis. Data on *medical and societal costs* are gathered using the TiC-P (Hakkaart-van Roijen et al. 2002). The TiC-P generates quantitative data about direct health care utilization (the type of care, its duration and medication) and indirect societal costs (cancer-related absence from work and cancer-related impairment in non-paid work). Unit cost estimates are derived from the national manual for cost prices in the health care sector (Hakkaart-van Roijen et al. 2010). Unit cost estimates are combined with resource utilization data to obtain a net cost per patient over the entire follow-up period. Unit cost estimates are derived from the national manual for cost prices in the health care sector. Costs of reduced ability to work are estimated using the friction costs method. Treatment costs are calculated using activity-based-costing methods, thus measuring actual resources (time of therapist, time of patients, facilities) used. Unit cost estimates are combined with resource utilization data to obtain a net cost per patient over the entire follow-up period.

To measure the *health-related quality of life* of cancer patients, a validated health-related quality of life instrument is used, the EuroQol-5D (EQ-5D; (The EuroQol Group 1990)). The EQ-5D is a generic instrument comprising five domains: mobility, self-care, usual activities, pain/discomfort and anxiety/depression. The EQ-5D index is obtained by applying predetermined weights to the five domains. This index gives a societal-based global quantification of the participant’s health status on a scale ranging from 0 (death) to 1 (perfect health). Participants are also asked to rate their overall quality of life on a visual analogue scale (EQ- 5D VAS) consisting of a vertical line ranging from 0 (worst imaginable health status) to 100 (best imaginable). The EQ-5D is available in a validated Dutch translation (Lamers et al. 2005). Because there are indications that the Short Form-12 (SF-12; (Ware et al. 1996)), another questionnaire on health-related quality of life, is more sensitive to change in populations with less severe morbidity than the EQ-5D (Johnson and Coons 1998), the SF-12 is administered as well. The SF-12 consists of 12 items yielding two summary scores for physical and mental health. Scoring is norm based with a mean of 50 (SD = 10); higher scores indicate better health.

## Effect predictors

Presence of DSM-IV Axis I mood/anxiety disorders is assessed by the SCID-I (First et al. 2012) which is a structured clinical interview. The interviewer rates answers on standardized questions during the interview on a scoring form. Subsequently, the presence or absence of symptoms is assessed. The SCID-I is administered by trained interviewers. An experienced psychiatrist (EBI) supervises the administration of the SCID-I. In the current study, neuroticism is assessed with the NEO Five Factor Inventory (Costa and McCrae 1992). A shorter version of the Revised NEO Personality Inventory (NEO-PI-R), the NEO-FFI has 60 items (12 per domain) derived from the original 240 items. The five factor domains assessed by this measure are neuroticism, extraversion, openness to experience, agreeableness, and conscientiousness. The psychometric properties of the Dutch NEO-FFI are good (Hoekstra et al. 1996).

## Process measures

*Mindfulness skills* are assessed with the 24-item Five Facet Mindfulness Questionnaire Short Form (FFMQ-SF). The FFMQ consists of five subscales: observing, describing, acting with awareness, non-judging of inner experience and non-reactivity to inner experience. The FFMQ is sensitive to change in mindfulness-based interventions (e.g. (Gu et al. 2015)). A Dutch 24-item short form of the FFMQ (FFMQ-SF) was developed and assessed in a sample of 376 adults with clinically relevant symptoms of depression and anxiety and cross-validated in an independent sample of patients with fibromyalgia (Bohlmeijer et al. 2011). The FFMQ-SF was positively related to well-being and openness to experience and inversely related to measures of psychological symptoms, experiential avoidance, and neuroticism.

In addition, in both group and internet-based MBCT the following process measures are administered at the start of each weekly session in order to determine processes of change during both interventions. In the group MBCT they are handed out in paper by the teacher, in the internet-based MBCT intervention they are provided online at the beginning of a new training week. The Mindful Attention Awareness Scale (MAAS; (Brown and Ryan 2003)) is administered weekly to assess *mindful attention in daily life*. The MAAS has been shown to have an similar factor structure in cancer patients as in the general population (Carlson and Brown 2005). Chronbach’s alpha for the Dutch version ranged between .82 and .87 (Schroevers et al. 2008). Positive and negative affect is assessed weekly using the International Positive and Negative Affect Scale - Short Form I-PANAS-SF). The crosscultural factorial invariance, internal reliability, temporal stability, and convergent and criterion-related validities of the I-PANAS-SF were found to be acceptable (Thompson 2007).

*Working alliance* is measured with a translated and shortened form of the Working Alliance Inventory (WAI; (Horvath and Greenberg 1989)), consisting of three subscales assessing: 1) how closely client and therapist agree on and are mutually engaged in the goals of treatment, 2) how closely client and therapist agree on how to reach the treatment goals, and 3) the degree of mutual trust, acceptance, and confidence between client and therapist. Patients score on a 5-point scale ranging from rarely to always (Stinckens et al. 2009; Hatcher and Gillaspy 2006). The 12-item inventory was validated in a Dutch-speaking sample and a recent study showed that internal consistency of the short form was > .80 for all separate subscales and .87 for the total (Janse et al. 2014). The WAI is administered before session 2, 5 and 9.

Self-reported *group cohesion* is assessed in the group MBCT condition with the Dutch Group Cohesion Questionnaire (GCQ) that has been used in cancer patients before (May et al. 2008). The GCQ consists of four subscales: the bond with the group as whole, the bond with other members, cooperation within the group and the instrumental value of the group bond. Each item is rated from 1 (totally disagree) to 6 (totally agree). Internal consistency of all scales was reported to range from adequate to good (0.66–0.88) (Trijsburg et al. 2004). The GCQ is administered before session 2, 5 and 9.

*Adherence* is assessed during the entire treatment period with a calendar (both for group and internet-based MBCT) on which participants fill out whether they adhere to both formal (e.g. the sitting meditation) and informal (e.g. 3-min breathing space) mindfulness exercises. Adherence to protocol has been shown to mediate the effects of MBCT on depressive symptoms [72].

### Semi-structured interviews

In order to more fully understand how interventions bring about change, it is important to complement quantitative research with qualitative research (Shennan et al. 2011). For this reason participants’ views on barriers and facilitators of the internet-based MBCT are explored in more detail by conducting semi-structured interviews in a purposive sample of participants in the trial.

### Statistical analysis

#### Sample size

Based on post treatment HADS scores within the routine outcome data of cancer patients who received mindfulness at the Helen Dowling Institute, we expected post treatment HADS scores of 10.6 (SD 6.4) in the MBCT interventions and 14.8 (SD 8.1) in the TAU condition. In the power calculation we ignored the dependency caused by the therapy groups, which has been found in previous research to be small (Van Aalderen et al. 2012). As we compare both group and internet-based MBCT to TAU, we corrected the corresponding alpha level to 0.025. Assuming a power of 0.9, a sample size of 65 per condition is needed. Taking an estimated expected dropout rate of 15 % in the group MBCT and TAU and 30 % in the internet-based MBCT into account, we aim to recruit 76 participants in the group MBCT and TAU conditions and 93 in the internet-based MBCT, thus 245 patients in total.

#### Statistical analysis

All analyses are carried out using the intention to treat and per protocol samples. The primary analysis is aimed at showing superiority of group MBCT and internet-based MBCT compared to TAU in terms of psychological distress directly post treatment in the intention to treat sample. Secondary analyses of the stability of the treatment effect are conducted using the data from the assessments at 3 and 9 months post-treatment, using linear mixed models to control for possible dependency caused by the repeated measurements.

We will use the bootstrapping procedure as it provides the most powerful and reasonable method of obtaining confidence limits for specific indirect effects under most conditions (Preacher and Hayes 2008). In all mediation analyses, post-treatment HADS scores are controlled for baseline HADS scores. Residual change scores for all potential mediators are calculated (MacKinnon 2008). To explore whether the mediators (partly) affect the relation of condition on post-treatment symptom levels, the model including the potential mediators is compared with the model without mediators for both univariate and multivariate models. Subsequently, 95 % bias corrected and accelerated confidence intervals (95 % CI) (Efron 1987) are calculated to explore the contribution of each individual mediator and the group of mediators in total.

#### Cost-effectiveness

The economic evaluation is based on the general principles of a cost-utility analysis and is performed alongside the clinical trial which compares three alternatives: 1) group MBCT; 2) internet-based MBCT, and 3) TAU. Primary outcome measures for the economic evaluation are: costs (here we follow the Dutch guidelines for costing research (Hakkaart-van Roijen et al. 2010)) and quality adjusted life years (QALY) measured by the EQ-5D. Secondary analyses will explore the possible differences in outcome with HrQoL measured by SF-12. The societal perspective is operationalized by including productivity losses/gains applying the friction cost method on a per patient basis by means of the TiC-P (Hakkaart-van Roijen et al. 2002).

The incremental cost-effectiveness ratio (ICER) “cost per Quality-Adjusted Life Year (QALY) gained” based on EQ-5D utilities according to the Dutch algorithm (Lamers et al. 2005) is computed and uncertainty surrounding these parameters is determined using the bootstrap method (dealing with potential skewness in the distributions). A cost-effectiveness acceptability curve will be derived that is able to evaluate efficiency by using a range of thresholds (Willingness To Pay for a QALY gained). The impact of uncertainty surrounding relevant deterministic parameters on the ICER is subsequently explored using one-way sensitivity analyses on the range of extremes.

The cost analysis exists of two main parts. First, on patient level, volumes of care is measured using patient questionnaires. Per arm (intervention and control groups) full cost-prices are determined using activity based costing. The second part of the cost analysis consists of determining the cost prices for each volume of consumption in order to use these for multiplying the volumes registered for each participating patient. The Dutch guidelines for cost analyses are used with regard to prices (Hakkaart-van Roijen et al. 2010). For units of care/resources where no guideline or standard prices are available real cost prices are determined.

## Discussion

A significant proportion of cancer patients suffers from psychological distress and is in need of appropriate psychological treatment (Mehnert et al. 2014). An increase in the number of patients who will have to deal with the consequences of having cancer is to be expected (KWF Kankerbestrijding 2011; Mitchell et al. 2011), which calls for more widely accessible and effective psychosocial treatment. Mindfulness-based treatment has proven to be effective in reducing psychological distress in cancer patients (Piet et al. 2012).

Providing internet-based mindfulness could hold promise in terms of increasing accessibility: patients do not have to travel and treatment planning is more flexible in the light of individual circumstances. Therefore, the current trial investigates the effectiveness in reducing psychological distress of both group- and internet-based MBCT compared to TAU.

Furthermore, although the need of cost-effectiveness evaluations of psycho-oncological interventions has long been recognized (Carlson and Bultz 2004), information on the cost-effectiveness of mindfulness interventions is largely absent. In addition to the clinical effectiveness, the current trial also investigates cost-effectiveness of both group- and internet-based MBCT interventions compared to TAU. We hope that our trial provides further insight into the accessibility, effectiveness and cost-effectiveness of group and internet-based MBCT in the reduction of psychological distress in patients with cancer.

## Abbreviations

TAU: Treatment as Usual
CBT: Cognitive behavioral therapy
ES: Effect size
MBCT: Mindfulness-Based Cognitive Therapy
MBSR: Mindfulness-Based Stress Reduction
HADS: Hospital Anxiety and Depression Scale
MAAS: Mindful attention and awareness scale
I-PANAS-SF: International positive and negative affect scale short form
SCID-I: Structural Clinical Interview for DSM-IV Axis I Disorders
TiC-P: Trimbos and iMTA questionnaire for Costs associated with Psychiatric illnesses
MBI-TAC: Mindfulness-Based Interventions – Teacher Assessment Criteria
FCRI: Fear of Cancer Recurrence Inventory
MHC-SF: Mental Health Continuum – Short Form
EQ-5D: EuroQol-5 Dimensions
SF-12: Short-Form-12
NEO-FFI: NEO-Five Factor Inventory
NEO-PI-R: NEO-Personality Inventory-Revised
FFMQ-SF: Five factor mindfulness questionnaire – short form
WAI: Working alliance inventory
GCQ: Group Cohesion Questionnaire
ICER: Incremental cost effectiveness ratio
QALY: Quality adjusted life year
